# Prediction of the Mechanical Properties of Basalt Fiber Reinforced High-Performance Concrete Using Machine Learning Techniques

**DOI:** 10.3390/ma15207165

**Published:** 2022-10-14

**Authors:** Ali Hasanzadeh, Nikolai Ivanovich Vatin, Mohammad Hematibahar, Makhmud Kharun, Issa Shooshpasha

**Affiliations:** 1 Department of Geotechnical Engineering, Babol Noshirvani University of Technology, P.O. Box 484, Babol 4714871167, Iran; 2 Peter the Great St. Petersburg Polytechnic University, 195251 St. Petersburg, Russia; 3 Department of Civil Engineering, Academy of Engineering, RUDN University, 6 Miklukho-Maklaya Street, 117198 Moscow, Russia; 4 Department of Reinforced Concrete and Stone Structures, Moscow State University of Civil Engineering, 26 Yaroslavskoye Highway, 129337 Moscow, Russia

**Keywords:** basalt fiber, high-performance concrete, mechanical properties, machine learning method

## Abstract

In this research, we present an efficient implementation of machine learning (ML) models that forecast the mechanical properties of basalt fiber-reinforced high-performance concrete (BFHPC). The objective of the present study was to predict compressive, flexural, and tensile strengths of BFHPC through ML techniques and propose some correlations between these properties. Moreover, the modulus of elasticity (ME) values and compressive stress–strain curves were simulated using ML techniques. In this regard, three predictive algorithms, including linear regression (LR), support vector regression (SVR), and polynomial regression (PR), were considered. LR, SVR, and PR were utilized to forecast the compressive, flexural, and tensile strengths of BFHPC, and the PR technique was employed to simulate the compressive stress–strain curves. The performance of the models was also determined by the coefficient of determination (R^2^), mean absolute errors (MAE), and root mean square errors (RMSE). According to the obtained values of R^2^, MAE, and RMSE, the performance of PR was better than other types of algorithms in estimating the compressive, tensile, and flexural strengths. For example, R^2^ values were 0.99, 0.94, and 0.98 in predicting the compressive, flexural, and tensile strengths using PR, respectively. This shows the higher accuracy and reliability of the PR technique compared with other predictive algorithms. Finally, we concluded that ML techniques can be appropriately applied to assess the mechanical characteristics of BFHPC.

## 1. Introduction

Basalt is a natural material that is found in volcanic rocks originating from frozen lava, with a melting temperature of 1500–1700 °C. The basalt fiber (BF) is produced by heating basalt rock in the furnace at 1450 to 1500 °C. The molten material is then passed through platinum and rhodium crucible bushing to form fiber. This technology is known as continuous spinning. The development of BF was first performed by the Moscow Research Institute of Glass and Plastic in 1953–1954, and its first industrial production furnace was completed in 1985 at a Ukraine fiber laboratory [1]. The fibers of basalt provide resistance against corrosion, heat, and chemical attack on the concrete, making them beneficial for use in building materials [2,3,4,5,6,7]. Previous studies have shown that BF is able to enhance the mechanical characteristics of concrete [8,9,10]. For example, Kharun et al. [11] found that the durability and stability of high-strength concrete (HSC) was increased after adding different percentages of BF. They found that BF improves flexural strength and prevents development of cracking in concrete blocks. Their results demonstrated that the optimum percentage of BF added to HSC is 1% (by concrete volume). With the addition of 1% BF, the compressive and tensile strengths of HSC increased more than 37 and 70%, respectively. Yang et al. [12] indicated that the addition of a proper amount of BF to ordinary concrete can delay initial cracking and increase the toughness of concrete. Long cracks on the surface of concrete gradually transform into many micro-cracks with increasing fiber content. They also reported that adding 0.6% BF can increase the compressive strength of concrete more than 13%. Biradar et al. [13] found that concrete containing 0.3% BF illustrated maximum strength values. They showed that the compressive, tensile, and flexural strength of concrete reinforced with 0.3% BF was 9.82, 36.7, and 18.83% more than their corresponding values in ordinary concrete, respectively. Katkhuda and Shatarat [14] reported that chopped BF had insignificant effects on the increase in compressive strength but considerably enhanced flexural and tensile strengths of recycled concrete aggregate.

High-performance concrete (HPC) has become very popular due durability and resistance against penetration of aggressive agents. HPC is widely used in structures such as tall buildings, bridges, runways, and highway pavements [15]. However, HPC is still considered a brittle material with poor tensile and flexural strengths. Therefore, it would be interesting to study the effect of BF on the mechanical properties of HPC. Ayub et al. [16,17] estimated the impact of chopped BF on the mechanical properties of HPC. Their results showed that adding 2% BF (by volume of the concrete) improved compressive strength more than 17%. They also mentioned the effect of BF on the enhancement of HPC ductility. Mohaghegh et al. [18] studied the impact of different percentages of BF on the characteristics of HPC and found that the highest flexural, tensile, and compressive strength values in HPC were achieved with the addition of 1.33% BF. According to their results, adding BF may be an effective way for enhancing the mechanical characteristics of HPC. Kharun et al. [19] investigated the effect of adding chopped BF on HPC characteristics. Their results proved that adding 1.2% BF had maximum effects on the improvement of mechanical characteristics. Thus, based on the results of previous investigations [16,17,18,19,20], the optimum percentage of BF to be added to HPC is usually more than 1% (by concrete volume).

On the other hand, machine; earning (ML), as a sub-field of artificial intelligence, has attracted attention in various industries, such as the medical, mechanical, and manufacturing industries [21,22,23]. ML has been greatly applied to the material sciences and civil engineering within the past decade, mainly for the prediction of the mechanical properties of different types of concretes [24,25,26,27,28,29,30,31,32,33,34,35,36]. Due to the effectiveness and capabilities of the modern computational processes, ML techniques can assess the mechanical properties of concrete without spending time in the laboratory, or investing money in experimentation [37]. For example, Su et al. [38] applied multiple linear regression (MLR), support vector machine (SVM), and artificial neural network (ANN) methods to forecast the mechanical characteristics of reinforced concrete with polymer fibers. They found that the developed SVM algorithm presented the best prediction results. In another example, Nguyen et al. [39] employed ML to find the best method for prediction of the compressive strength of geo-polymer reinforced concrete, out of 335 mix proportions. Sami Ullah et al. [40] applied support vector regression (SVR) and random forest (RF) techniques for strength assessment of lightweight foam concrete (LFC). They depicted that acceptable accuracy in the prediction of compressive strength of LFC was obtained by employing the RF. According to their findings, the coefficient of determination (R^2^) value was 0.95 via RF. They concluded that the RF method was able to predict compressive strength with high accuracy. Liu [41] investigated the prediction of the mechanical properties of HPC using extreme gradient boosting (XGBoost), SVR, and RF. He showed that the XGBoost algorithm has appropriate performance in predicting the compressive strength of HPC. The R^2^ > 0.99 was indicative of the high accuracy of his model in the prediction. Kashyzadeh et al. [42] utilized a back-propagation neural network (BPNN) optimized by a genetic algorithm (GA) to predict the compressive strength of concrete. Their objective was to find the predictive results of concrete strength via analyzing the curing temperature and the shape and size of aggregates as the most important variables. They illustrated that the developed neural network methods were consistent with the experimental ones.

Although researchers prefer to predict the mechanical properties of concrete through ML, their research fields have mainly concentrated on predicting some limited characteristics, such as compressive strength. They did not consider other mechanical properties, such as flexural and tensile strengths, which play important roles in concrete behavior. In the present paper, not only the compressive strength, but also the flexural and tensile strengths of concrete were predicted through three ML techniques, including linear regression (LR), SVR, and polynomial regression (PR). Then, compressive stress–strain curves were simulated through PR. Moreover, the values of the modulus of elasticity (ME) were estimated using ML techniques and compared with the relations existing in the literature. Finally, some correlations were proposed between the compressive, flexural, and tensile strengths.

## 2. Methods and Materials

### 2.1. Methods

ML provides systems with the ability to automatically learn and improve processes from experience without being explicitly programmed. According to the nature of input that is provided to a ML algorithm, ML can be classified into four different classes [43]:

- Supervised Learning (SL)
- Unsupervised Learning (USL)
- Semi-Supervised Learning (SSL)
- Reinforcement Learning (RL)

SL is the most common type of ML algorithm and it was used in this study to forecast the mechanical properties of BFHPC. SL seeks to create a model by discovering relationships between the input and output data and then makes predictions of the response values for a new dataset. Unlike the SL, USL is self-organized learning, and only input data is provided to the model in USL. SSL is between the SL and USL families. SSL uses both labeled and unlabeled data for training. Trial error search and delayed reward are the most relevant features of RL. RL permits the automatic determination of the ideal behavior within a specific context to maximize desired performance.

Recently, Python has become the most popular data science and ML programming language. Hence, in this study, three ML models including LR, SVR, and PR were run with Python code utilizing Anaconda software. Spyder was selected from the Anaconda navigator for model execution. “Sklearn”, an open-source ML library for Python, was also used for predictive data analysis.

In this study, three ML models, including LR, SVR, and PR, were selected to predict the mechanical properties of BFHPC. The LR model codes were programmed via the “LinearRegression” command in the “sklearn” Python packages. The linear equation is defined as [44]:

(1) y=ax+b

where *x* and *y* are variables, and *a* and *b* are the slope and intercept coefficient, respectively.

The support vector machine (SVM) is a well-known SL model which was developed in the 1990s. SVM is a complex ML algorithm that was applied by investigators to solve challenging engineering problems, such as forecasting. When SVM is used in regression applications, it is called SVR [45]. Unlike LR, SVR allows non-linear fitting for problems as well. SVR predicts values by minimizing errors and creating a separate line between data to fit the actual values.

In PR, the following polynomial function (Equation (2)) is employed to fit non-linear data [46]:

(2) $$y_{\left(x\right)}=a_{n}x^{n}+a_{n-1}x^{n-1}+a_{n-2}x^{n-3}+\ldots +a_{1}x^{1}+a_{0}$$

where *a* is the coefficient of a polynomial function, *a*_0_ is the Y-intercept of polynomial function or a constant value, *x* is a variable, and *y*_(*x*)_ is a variable dependent to *x*.

To scrutinize the quality of each model presented in this research, a set of three indicators were taken into account, including coefficient of determination (*R*^2^), mean absolute error (*MAE*), and root mean squared error (*RMSE*).

The *R*^2^ is a measure utilized in analysis to evaluate how well a model predicts future outcomes. It is shown in Equation (3) [47]:

(3) $$R^{2}=1-\frac{\sum_{}^{n}{\left(y-\hat{y}\right)}^{2}}{\sum_{}^{n}{\left(y-\overset{-}{y}\right)}^{2}}$$

where *y*, $\hat{y}$, and $\overset{-}{y}$ are the actual, predicted, and mean of the actual value, respectively. As seen in Equation (4), *MAE* is equal to the sum of the numerical differences of the values of community set divided by whole numbers (*n*). It calculates the average error utilizing the absolute difference from the actual data and he predicted results. *MAE* is found using Equation (4) [47]:

(4) $$MAE=\frac{1}{n}\sum_{}^{n}\left|y-\hat{y}\right|$$

*RMSE* measures the average deviation of each actual data point and the predicted results. It is obtained through Equation (5) [47]:

(5) $$RMSE=\sqrt{\frac{1}{n}\sum_{}^{n}{\left(y-\hat{y}\right)}^{2}}$$

### 2.2. Materials and Sample Preparation

To provide BFHPC samples, ordinary Portland cement, silica fume, tap water, superplasticizer, quartz sand (as fine aggregates), crushed granite (as coarse aggregates), and BF were used. Table 1, Table 2 and Table 3 present properties of cement, silica fume, and BF, respectively. Table 4 presents the mixture design of BFHPC in this study. As seen, BF percentage was the only variable factor in the mixture design. It should be noted that the initial and final setting time of the cement was 60 and 600 min, respectively. Silica fume, with a bulk density of 152.2 kg/m^3^, was used to fill the empty spaces between the BF and cementitious matrix. The superplasticizer was also added to reduce water consumption and increase workability of concrete. To mix materials, a laboratory concrete pan mixer was applied at the constant rate of 48 rpm. First, aggregates were mixed with each other for about 2 min. Then, water, cement, silica fume, and superplasticizer were added and mixed. Finally, fibers were manually inserted at a rate of 10 g per second and blended until a homogeneous mix was achieved. After that, a set of samples, including cylindrical samples, with diameters of 150 mm and height of 300 mm for tensile tests, cubic samples with dimensions of 100 × 100 × 100 mm^3^ for compressive tests, and prismatic samples with dimensions of 400 × 100 × 100 mm^3^ for flexural tests were prepared. To cure concrete specimens, they were kept under water at s temperature of approximately 20 °C. After curing for 28 days, samples were tested for determination of their compressive, flexural, and tensile strength values according to GOST10180 [48], ASTM C293/C293M [49], and ASTM C496 [50], respectively.

## 3. Results and Discussion

### 3.1. Experimental Results

**Figure 1:**
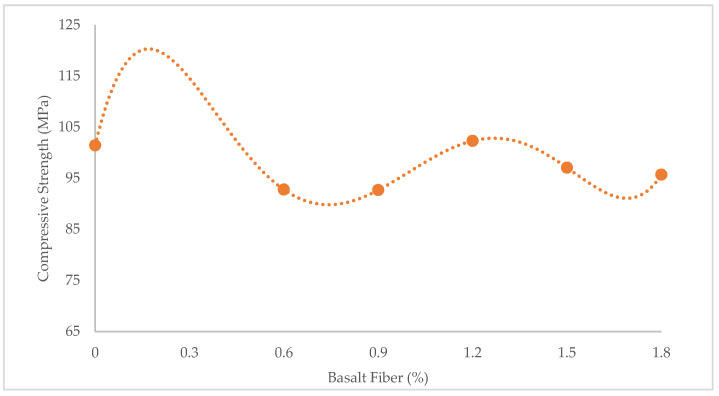
Variation of compressive strength with BF.

**Figure 2:**
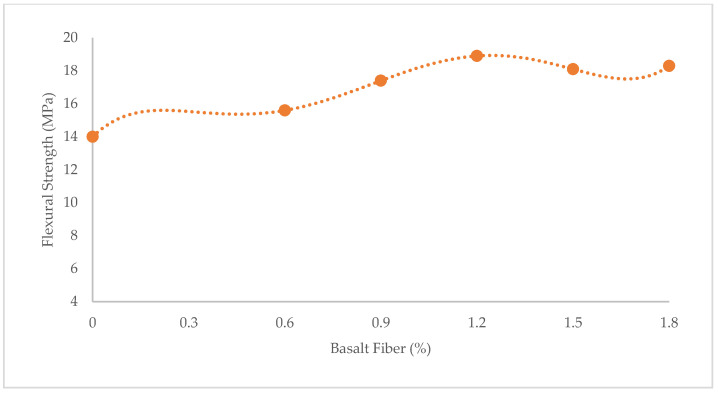
Variation of flexural strength with BF.

**Figure 3:**
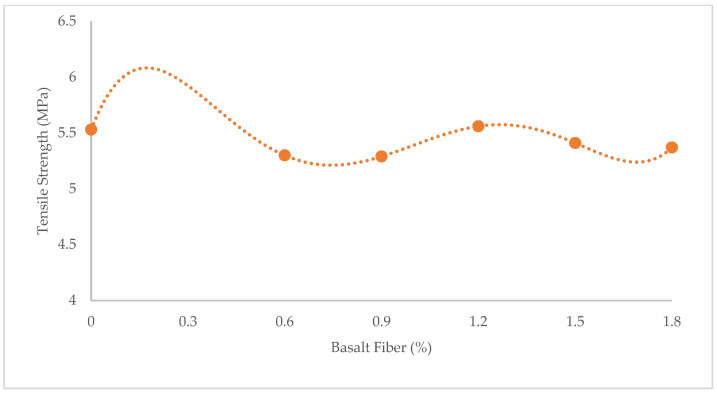
Variation of tensile strength with BF.

Figure 1, Figure 2 and Figure 3 show variations in the compressive, flexural, and tensile strength of BFHPC with BF, respectively. It was found that the optimum percentage of chopped BF added to HPC was 1.2%. This similarity observed between compressive and tensile behaviors of concrete (Figure 1 and Figure 3), although the flexural strength showed a different trend (Figure 2). As seen, the flexural strength smoothly increased by adding more chopped BF. In fact, BF affected micro-cracks and prevented their propagation in the samples. The inclusion of BF into cementitious composites improved the flexural strength by bridging the components in the composites and increasing the energy absorption. Furthermore, the higher bond and friction between BF and concrete matrix, leading to the improvement of the flexural strength [20,51].

**Figure 4:**
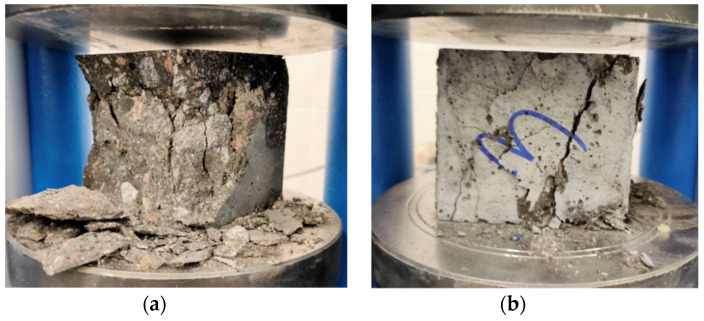
Failure mode of cubic samples: (**a**) HPC and (**b**) BFHPC1.2.

The effect of BF on the compressive and the strengths did not always show improving trends, which wasconsistent with the results found by Ayub et al. [16,17] and Mohaghegh et al. [18]. Although fibers of basalt did not significantly increase the compressive strength, they changed the failure mode of the samples. The fiber-reinforced samples failed smoothly during tests, i.e., they showed ductile behavior. This was due to the bridging effect of fibers which effectively hindered the further creation of cracks in the samples [52,53]. However, the unreinforced concrete samples failed suddenly. In other words, their failure mode was brittle (Figure 4).

### 3.2. Prediction Results

In this section, the mechanical properties of BFHPC were predicted using LR, SVR, and PR. Moreover, MAE, RMSE, and R^2^ are used for evaluation of prediction accuracy. It should be noted that the R^2^ values were between 0 and 1. The higher value of R^2^ meant better accuracy in prediction. The lower values of MAE and RMSE indicated that the prediction was closer to the experimental data. The required information for ML was gathered from the literature [1,3,4,5,8,9,12,13,16,17,18].

#### 3.2.1. Prediction of Compressive Strength

**Figure 5:**
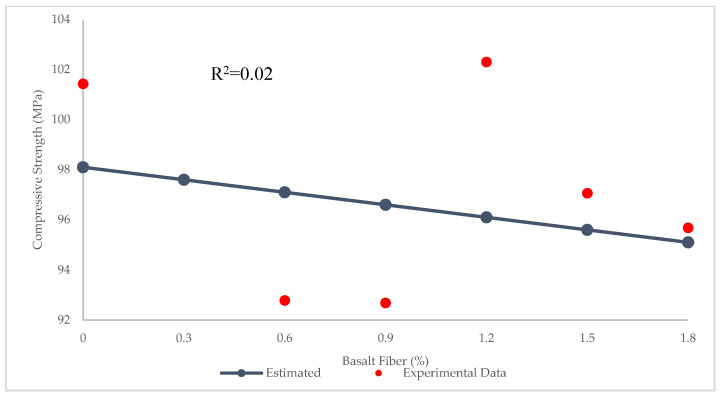
Comparison of experimental and predicted compressive strength values using LR.

**Figure 6:**
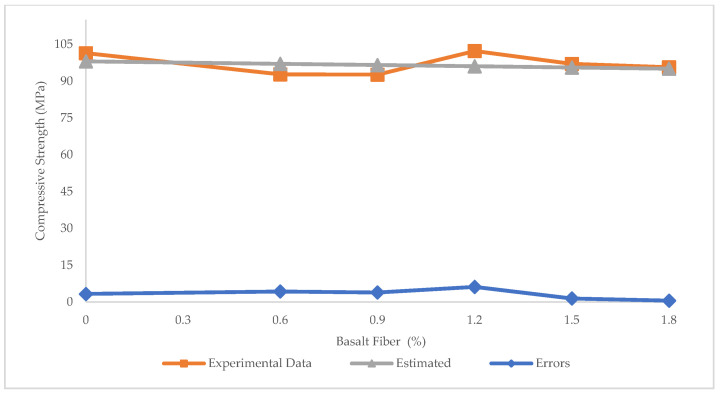
Experimental and estimated values and error distribution for compressive strength using LR.

In many previous studies, LR was used to predict the compressive strength of concrete, because it is the most basic and easily applied method. Figure 5 depicts the experimental and predicted values of compressive strength for BFHPC via LR technique. The R^2^ = 0.02 illustrates that LR performs poorly in predicting the compressive strength of BFHPC. As observed in Figure 6, the maximum and minimum errors between experimental and estimated results were 6.2 and 0.58 MPa, respectively. In addition, RMSE and MAE values were 13.9 and 3.17 MPa, respectively.

**Figure 7:**
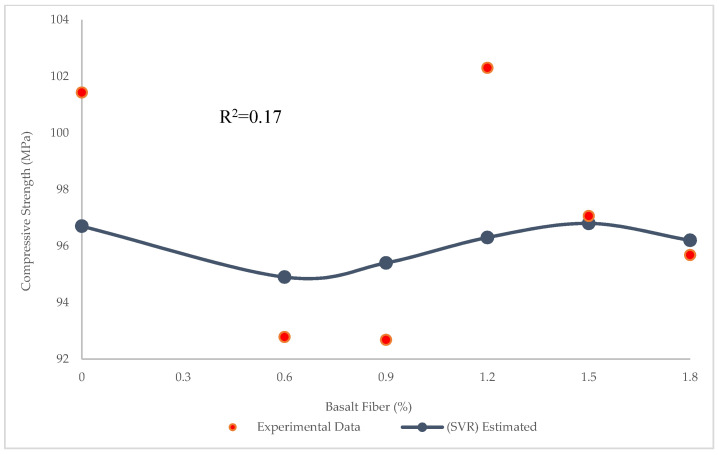
Comparison of experimental and predicted compressive strength values using SVR.

**Figure 8:**
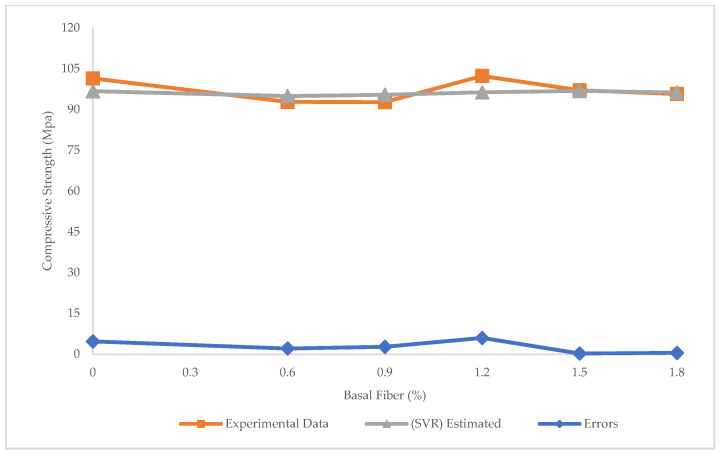
Experimental and estimated values and error distribution for compressive strength using SVR.

Figure 7 demonstrates the prediction of compressive strength values through the SVR. The R^2^ = 0.17 in this model shows that SVR performed better than LR in predicting compressive strength. However, the R^2^ values were still not satisfactory for both SVR and LR. As shown in Figure 8, the maximum and minimum errors between experimental and estimated results were 6.0 and 0.26 MPa, respectively. Furthermore, RMSE and MAE values obtained were 11.6 and 2.7 MPa, respectively. Although both SVR and LR showed many errors, the SVR was better than LR in the prediction of compressive strength.

**Figure 9:**
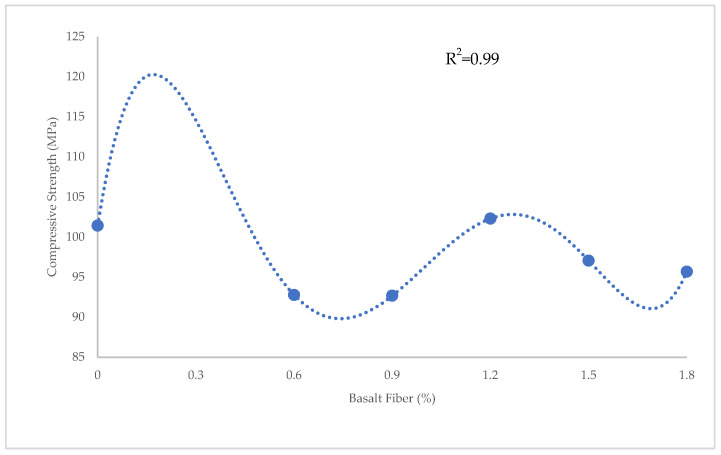
Comparison of experimental and predicted compressive strength using PR.

**Figure 10:**
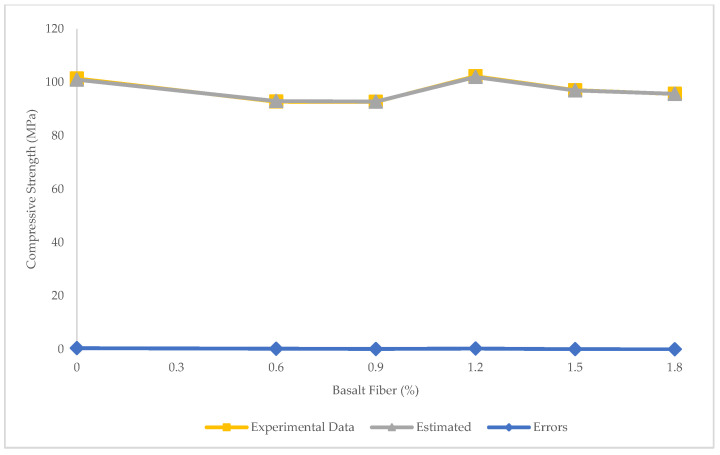
Experimental and estimated values and error distribution for compressive strength using PR.

Figure 9 shows the prediction of compressive strength for BFHPC samples using PR. In PR, the relationship between compressive strength and BF percentage was found by applying the data mining between inputs and outputs. The R^2^ = 0.99 shows that PR performed better than LR and SVR in predicting the compressive strength. Moreover, the maximum error of 0.43 MPa, minimum error of 0.02 MPa, RMSE of 0.05 Mpa, and MAE of 0.19 MPa confirmed that PR presented the most precise results (Figure 10). The results obtained using the PR technique for predicting the compressive strength in this study were consistent with the results of Kumar et al. [54]. They used different ML techniques, such as gaussian process regression (GPR), support vector machine regression (SVMR), and ensemble learning (EL) to estimate the compressive strength of lightweight-concrete. Based on their findings, GPR presented the most reliable results with an R^2^ of greater than 0.98. Their model was suitable for forecasting the compressive strength of concrete. However, other mechanical properties of concrete, such as flexural and tensile strengths, were not predicted in their analysis. The results of the prediction of compressive strength using GPR in this study (R^2^ > 0.98) was close to the PR results of the current investigation (R^2^ = 0.99).

Table 5 presents the comparison of experimental and predicted compressive strength values. As shown, PR predictions were more accurate and reliable than other algorithms. In fact, PR could closely predict the compressive strength to the experimental results.

#### 3.2.2. Prediction of Flexural Strength

**Figure 11:**
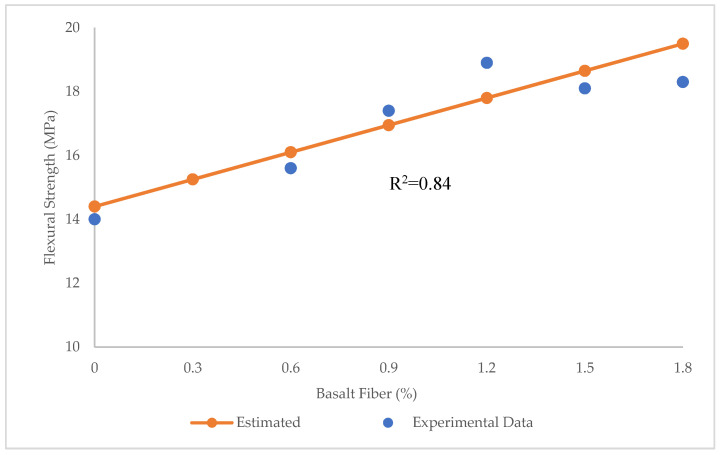
Comparison of experimental and predicted flexural strength values using LR.

Figure 11 depicts the prediction of flexural strength through LR. The R^2^ = 0.84 in this model exhibited that the LR was quite precise for predicting the flexural strength. In other words, LR technique could forecast values of flexural strengths close to experimental ones.

**Figure 12:**
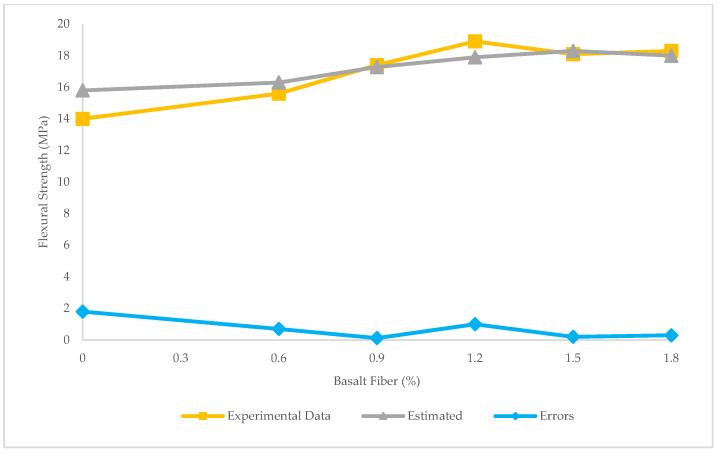
Experimental, estimated, and error values for flexural strength using LR.

As observed in Figure 12, the maximum and minimum error between experimental and estimated results was 1.1 and 0.45 MPa, respectively. In addition, RMSE and MAE values were obtained 0.54 and 0.64 MPa, respectively.

**Figure 13:**
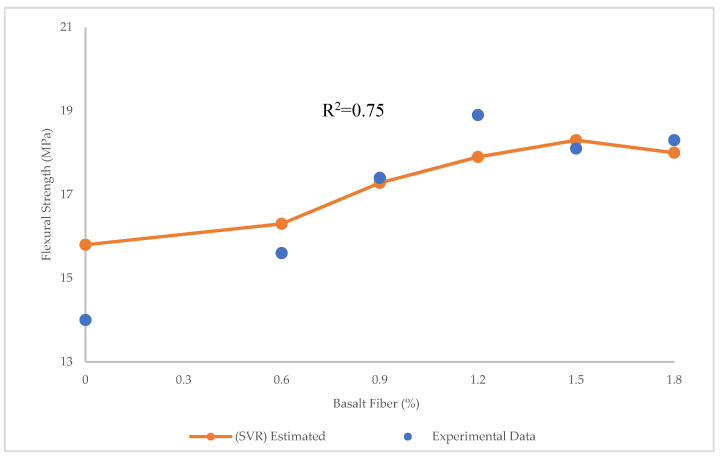
Comparison of experimental and predicted flexural strength values using SVR.

**Figure 14:**
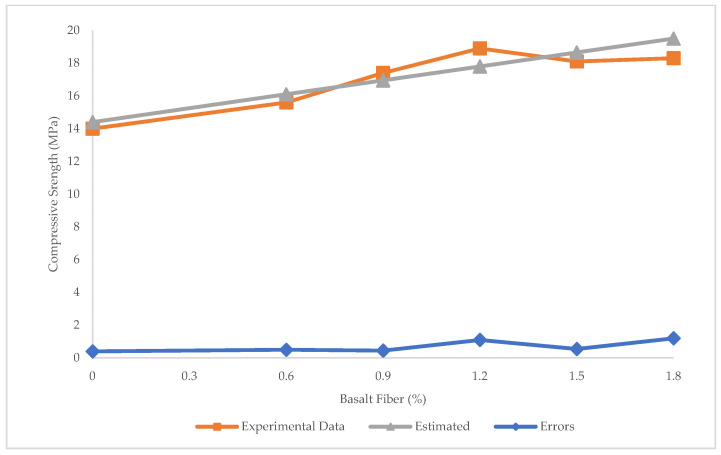
Experimental, estimated, and error values for flexural strength using SVR.

The comparison of the experimental and estimated results for flexural strength using SVR is shown in Figure 13. The R^2^ = 0.75 in SVR shows that this model is not accurate enough to predict the flexural strength. Figure 14 illustrates that the maximum and minimum error between experimental and predicted values through SVR is 1.8 and 0.12 MPa, respectively. The values of RMSE = 0.73 MPa and MAE = 0.63 MPa in SVR also confirm that the SVR is not as accurate as LR in the prediction of flexural strength.

**Figure 15:**
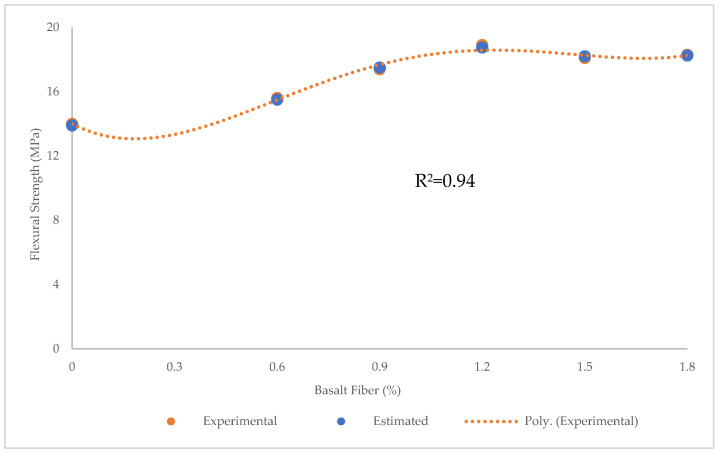
Comparison of experimental and predicted flexural strength values using PR.

**Figure 16:**
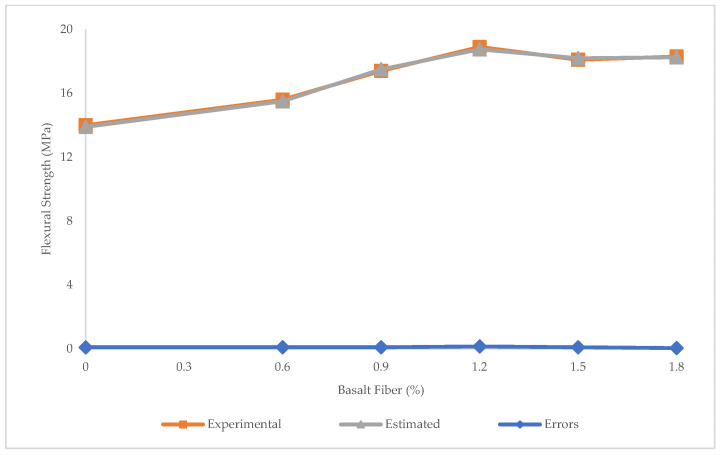
Experimental and estimated values and error distribution for flexural strength using PR.

The values of R^2^ = 0.94, RMSE = 0.025 MPa, MAE = 0.017 MPa, maximum error = 0.1 Mpa, and minimum error = 0.05 MPa obtained using PR showed that this algorithm was better than LR and SVR in predicting the flexural strength of BFHPC (Figure 15 and Figure 16).

Several researchers, such as Zheng et al. [55], forecasted the flexural strength of concrete using ML approaches. They predicted the flexural strength of steel fiber-reinforced concrete (SFRC) via RF, gradient boosting (GB), and XGBoost. Based on their findings, the GB model produced more precise results, with an R^2^ value of 0.96. The R^2^ values of RF and XGBoost models were 0.81 and 0.87 in their study, respectively.

Table 6 presents the comparison of experimental and predicted flexural strength values in this study. As the values predicted by PR were closer to the experimental ones, this suggests PR had the best performance in the prediction of flexural strength among the other ML models.

#### 3.2.3. Prediction of Tensile Strength

**Figure 17:**
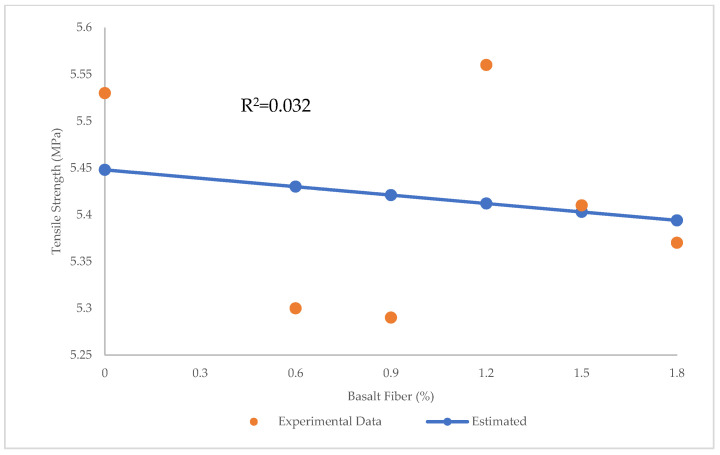
Comparison of experimental and predicted tensile strength values using LR.

**Figure 18:**
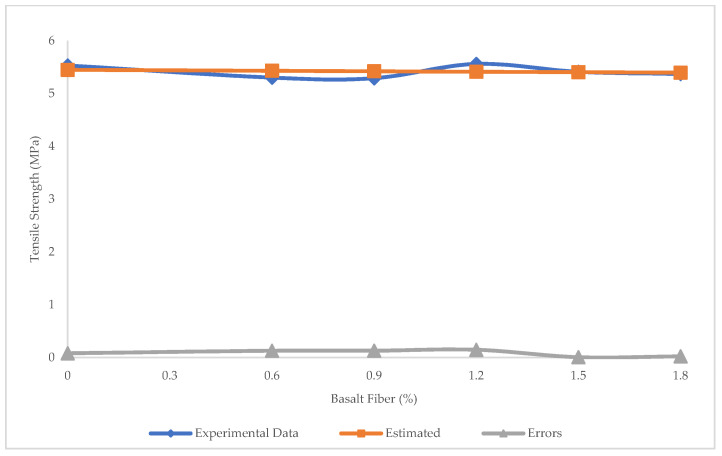
Experimental and estimated values and error distribution for tensile strength using LR.

Figure 17 shows the prediction of tensile strength of BFHPC using LR. The R^2^ = 0.032, RMSE = 0.0104 Mpa, and MAE = 0.086 MPa confirm that LR was unable to make reasonable predictions of tensile strength. Figure 18 illustrates the errors between predicted and experimental values of tensile strength via LR. The minimum error was 0.007 MPa and maximum error was 0.148 MPa in LR prediction.

**Figure 19:**
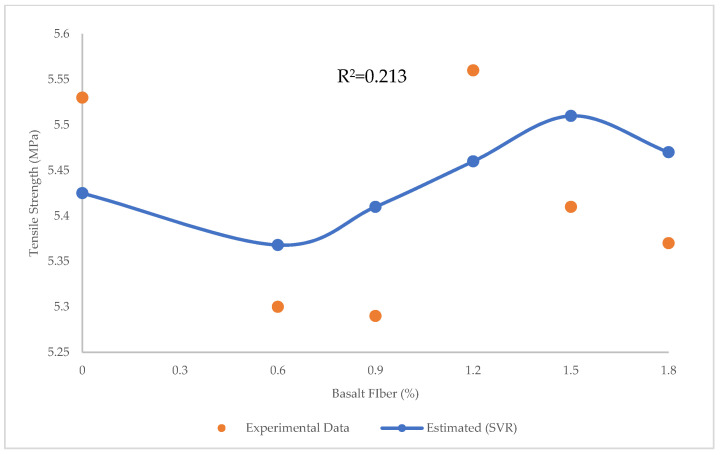
Comparison of experimental and predicted tensile strength values using SVR.

**Figure 20:**
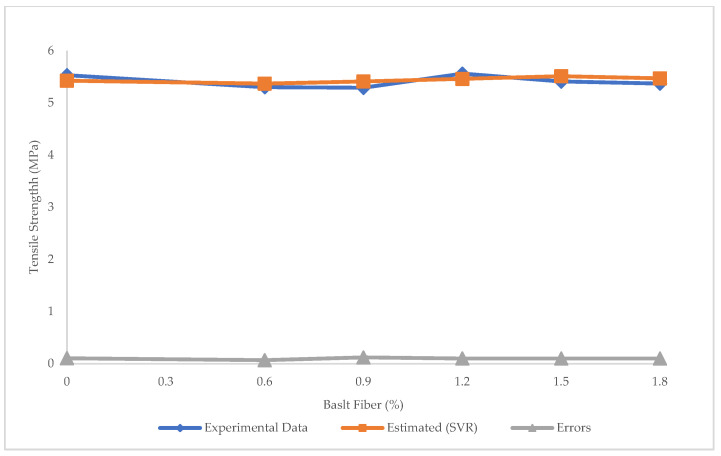
Experimental and estimated values and error distribution for tensile strength using SVR.

Figure 19 and Figure 20 indicate the prediction of tensile strength using SVR, and the errors between experimental and estimated results using SVR, respectively. The R^2^ = 0.213, RMSE = 0.01 Mpa, and MAE = 0.098 MPa in SVR, showing that though there were many errors in both LR and SVR, SVR was more precise than LR in the prediction of tensile strength. The maximum and minimum error between experimental and predicted tensile strength values using SVR was 0.12 and 0.068 MPa, respectively.

**Figure 21:**
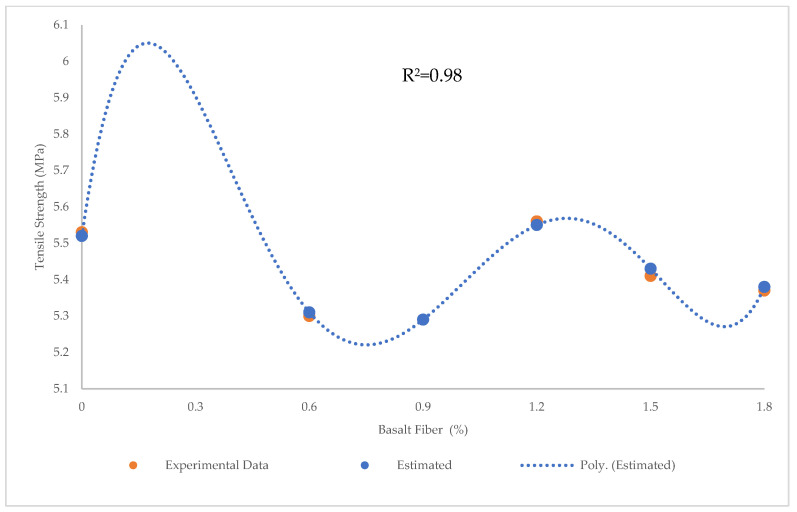
Comparison of experimental and predicted tensile strength values using PR.

**Figure 22:**
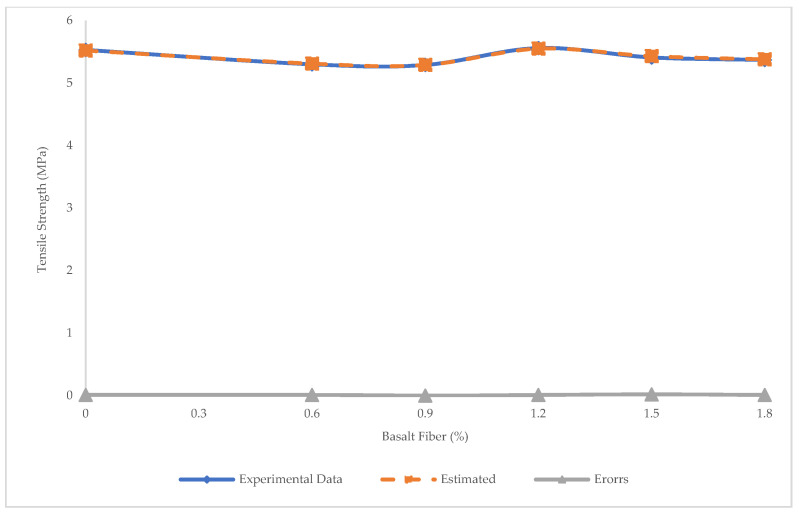
Experimental and estimated values and error distribution for tensile strength using PR.

Tensile strength was also predicted using the PR algorithm. The R^2^ = 0.98, RMSE = 0.05 MPa, and MAE = 0.19 MPa using this algorithm shows that PR was more accurate than LR and SVR in predicting tensile strength (Figure 21). Experimental and estimated values and error distribution for PR (Figure 22) showed that the maximum and minimum errors were 0.002 and 0 MPa, respectively.

Pan et al. [56] used ML algorithms, including RF, AdaBoost, and Bagging, for prediction of the tensile strength of recycled aggregate concrete (RAC). They found that the RF with R^2^ = 0.96 and low error (RMSE = 0.49 MPa) had superior performance in prediction among the other algorithms. Table 7 presents the comparison of experimental and estimated tensile strength values. As observed, PR generally had more accurate and meaningful predictions.

### 3.3. Prediction of Stress–Strain Curves and Modulus of Elasticity

**Figure 23:**
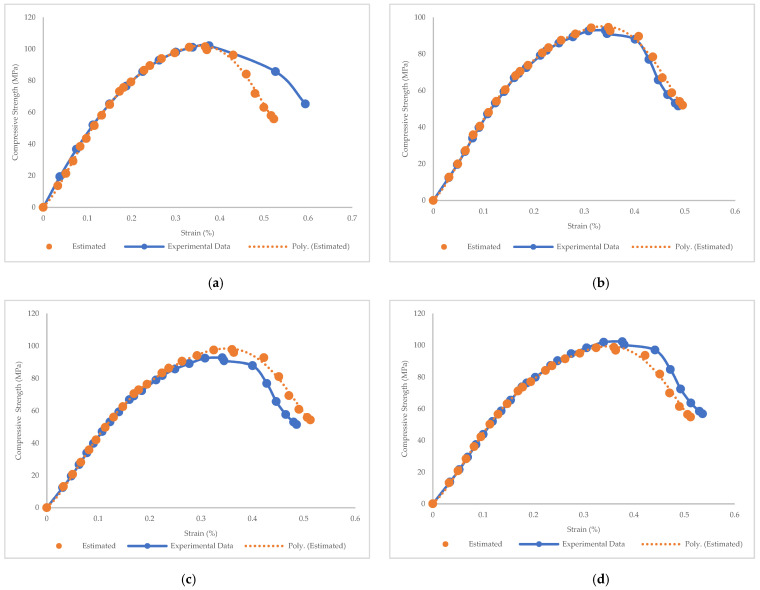

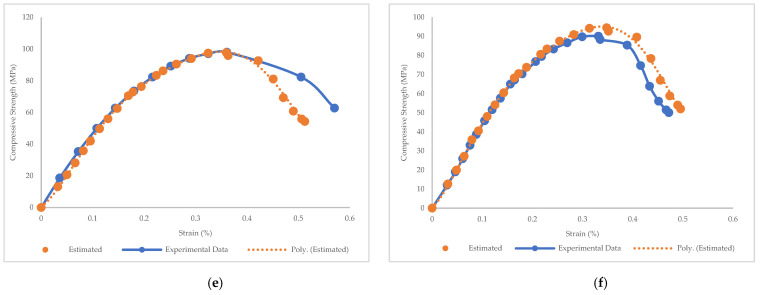
Prediction of stress-strain curves through PR: (**a**) HPC, (**b**) BFHPC0.6, (**c**) BFHPC0.9, (**d**) BFHPC1.2, (**e**) BFHPC1.5, and (**f**) BFHPC1.8.

In this study, PR was not only used to forecast the mechanical properties of BFHPC, but it was also applied to simulate the compressive stress–strain curves. The relationship between compressive strength and BF percentage made it possible to plot stress–strain curves of BFHPC. The prediction at each point was carried out using the polynomial function (Equation (2)), and then, the stress–strain curves were simulated by fitting curves through the prediction points. Figure 23 demonstrates the experimental and predicted stress–strain curves for HPC, BFHPC.06, BFHPC0.9, BFHPC1.2, BFHPC1.5, and BFHPC1.8. As observed, compressive stress–strain curves were accurately simulated. In fact, the PR algorithm was not only able to predict the elastic and hardening phase, but it also simulated the plastic phase. Due to the linear behavior in the elastic phase, the prediction of this part of the compressive stress–strain curves was easy. However, simulation of the plastic phase of curves had more challenges. The PR algorithm appropriately simulated the plastic phase of compressive stress–strain curves as well as the elastic phase, by fitting prediction points.

A review of previous studies showed that there has been a lack of simulations of compressive stress–strain curves. For example, Carreira and Chu [57] presented a model to predict compressive stress–strain curves. However, they simulated the curves by developing a mathematical model that only focused on the elastic phase of compressive stress–strain curves. They did not consider the plastic phase. In another example, Ezeldin and Balaguru [58] provided a method to estimate the compressive stress–strain curve. However, they used complex prediction formulas in their method. In the present research, the compressive stress–strain curves of BFHPC samples were estimated using ML (not mathematical) techniques that considered the plastic phase. As shown, the curves were simulated with high accuracy.

The modulus of elasticity (*ME*) is one of the most important properties of concrete because it shows the concrete capacity to resist deformation under applied load. Researchers have suggested different relations for estimating *ME* using the compressive strength of concrete (*f′_c_*). Equations (6)–(9) present some of these relations available in the literature (ACI 318-08 [59], Gardner and Lockman [60], Eurocode [61], CEB-FIP [62]):

(6) $$ME=4743\sqrt{{f^{\prime }}_{c}}$$

(7) $$ME=3500+4300\sqrt{{f^{\prime }}_{c}}$$

(8) $$ME=22000\left(0.1\sqrt{{f^{\prime }}_{c}}\right)$$

(9) $$ME=9980\sqrt[{3}]{{f^{\prime }}_{c}}$$

Table 8 demonstrates the comparison of *ME* values obtained by our experiments, predictions, and formulas from the literature. As seen, the ME values found using ACI 318-08 [59], Eurocode [61], and PR were closest to the experimental ones.

### 3.4. Relationship between Compressive, Flexural, and Tensile Strengths

In this section, the relationship between compressive, flexural, and tensile strengths of BFHPC samples was investigated. With the aid of these relationships, the results of one test could be assessed using the results of another test. Thus, there would be no need to conduct different tests to obtain the required results. To obtain the relationship between compressive and flexural strengths of BFHPC, the parabolic function was used in this research. For this goal, the parabolic function was used more than five times to find the best relationship, and as a result, Equation (10) was obtained:

(10) $${f^{\prime }}_{c}=1.6{\left({f^{\prime }}_{f}-16.5\right)}^{2}+91.8$$

where *f’_c_* and *f’_f_* is the compressive and flexural strength, respectively.

**Figure 24:**
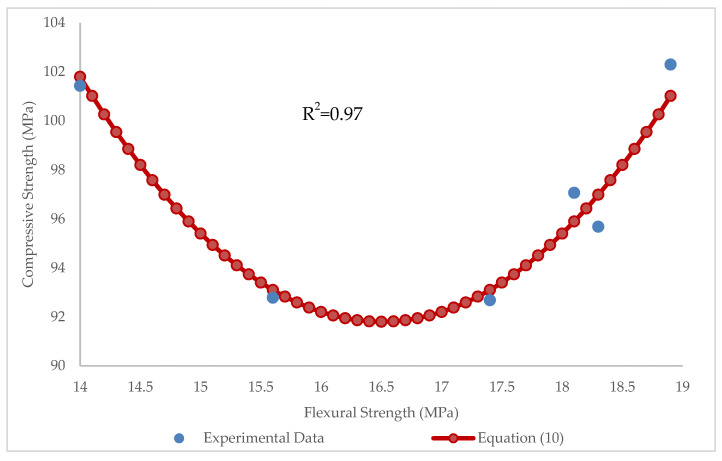
Relationship between compressive and flexural strengths.

Considering the values of R^2^ = 0.97, RMSE = 0.42 MPa and MAE = 0.43 MPa, we can conclude that there is an acceptable relationship between compressive and flexural strengths (Figure 24).

**Figure 25:**
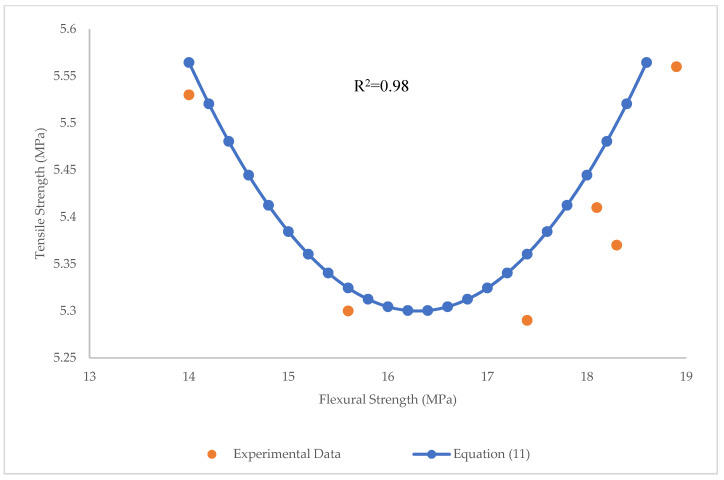
Relationship between tensile and flexural strengths.

The relationship between flexural (*f’_f_*) and tensile strength (*f’_t_*) was found by a parabolic function (Equation (11)) and is depicted in Figure 25:

(11) $${f^{\prime }}_{t}=0.045{\left({f^{\prime }}_{f}-16.6\right)}^{2}+5.28$$

**Figure 26:**
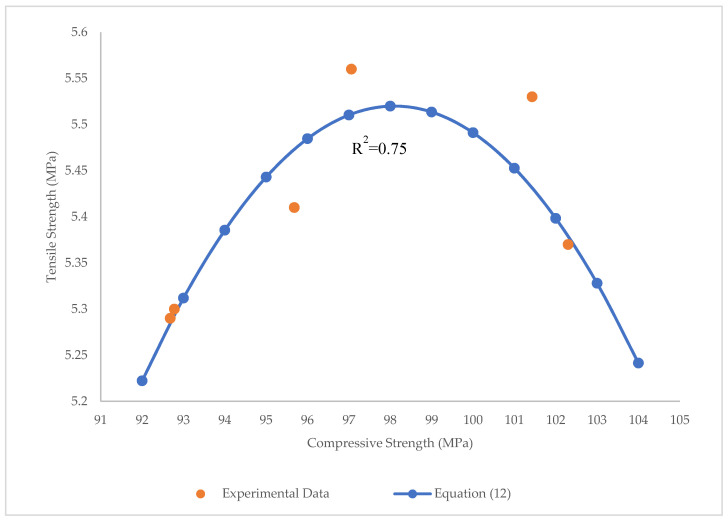
Relationship between tensile and compressive strengths.

The high value of R^2^ = 0.98 and low values of RMSE and MAE (RMSE = 0.0012 MPa, MAE = 0.026 MPa) show that the parabolic function is an appropriate function to correlate the tensile and flexural strengths of BFHPC. Furthermore, using the parabolic function, the correlation between compressive and tensile strength of BFHPC was found (Equation (12)), and is indicated in Figure 26:

(12) $${f^{\prime }}_{t}=0.008{\left({f^{\prime }}_{c}-98.1\right)}^{2}+5.52$$

The R^2^ = 0.75, RMSE = 0.0027 Mpa, and MAE = 0.034 MPa indicate that there is a good relation between these parameters. As seen, parabolic functions could present appropriate correlations between the compressive, tensile, and flexural strengths of BFHPC. The relationship between tensile and flexural strengths with R^2^ = 0.98 was very strong among the others. The compressive, flexural, and tensile strengths of BFHPC can only be determined through continuous laboratory experiments, which are time-consuming and needs a greater workforce. This issue could be significantly solved by using these proposed relations.

## 4. Conclusions

In this research, ML techniques were applied for the prediction of the different mechanical properties of BFHPC. For this purpose, three predictive algorithms, LR, SVR, and PR, were employed and analyzed to find the most accurate predictions. Moreover, the compressive stress–strain curves of samples were simulated by fitting through the prediction points. The prediction of the modulus of elasticity (ME) and comparison of forecasted ME with the relations available in the literature was also part of this research. Finally, some correlations between compressive, tensile, and flexural strengths of BFHPC were suggested. The following results can be drawn from this investigation:

- The mechanical characteristics of BFHPC can be more accurately predicted via PR in comparison with LR and SVR. For example, in predicting the compressive strength through PR, the values of R^2^, RMSE, and MAE were 0.99, 0.05 Mpa, and 0.19 MPa, respectively. This confirms the high accuracy of PR in terms of its prediction.
- Although simulation of compressive stress–strain curves has challenges (particularly simulation of the plastic phase), the PR technique was able to appropriately forecast these curves.
- The predicted values of ME, one the most important properties of concrete, using PR were close to the experimental results and results of some available formulas in the literature.
- Proposed models could be efficiently used at the construction site to minimize required laboratory work, as well as save time and costs.

More reliable and high-quality experimental data will play a vital role in improving the model performance. In other words, a database and more input parameters may be required for generation of a better response from employed models in future. ML techniques may be utilized with heuristic approaches, such as the whale optimization algorithm, ant colony optimization, and particle swarm optimization, to achieve more effective and precise outcomes. Future studies should compare these tactics with current findings.

## Figures and Tables

**Table 1:** Properties of ordinary Portland cement.

Oxide (%)								Blaine (m^2^/kg)	Specific Gravity
SiO_2_	Fe_2_O_3_	MgO	SO_3_	Al_2_O_3_	CaO	K_2_O	L.O.I.		
19.52	4.04	4.36	2.89	4.81	62.18	0.6	1.62	387	3.14

**Table 2:** Chemical composition of silica fume.

Chemical Composition	Value (%)
Silicon dioxide (SiO_2_)	90–92
Alumina (Al_2_O_3_)	0.68
Iron oxide (Fe_2_O_3_)	0.69
Calcium oxide (CaO)	1.58
Magnesium oxide (MgO)	1.01
Sodium oxide (Na_2_O)	0.61
Potassium oxide (K_2_O)	1.23
Carbon (C)	0.98
Sulfur (S)	0.26

**Table 3:** Properties of chopped basalt fibers.

Length (mm)	Diameter (µm)	Tensile Strength (MPa)	Young’s Modulus (GPa)	Elongation (%)	Specific Gravity
18	17.9	4100–4840	93.1–110	3.1	2.63–2.8

**Table 4:** Mixture design of BFHPC.

Sample Name	Cement (kg/m^3^)	Silica Fume (kg/m^3^)	Quartz Sand (kg/m^3^)	Crushed Granite (kg/m^3^)	Superplasticizer (kg/m^3^)	Water (kg/m^3^)	BF (% of Concrete Volume)
HPC	500	125	585	1005	12.5	187.5	0
BFHPC0.6	500	125	585	1005	12.5	187.5	0.6
BFHPC0.9	500	125	585	1005	12.5	187.5	0.9
BFHPC1.2	500	125	585	1005	12.5	187.5	1.2
BFHPC1.5	500	125	585	1005	12.5	187.5	1.5
BFHPC1.8	500	125	585	1005	12.5	187.5	1.8

**Table 5:** The experimental and estimated compressive strength values (MPa).

Compressive Strength	HPC	BFHPC0.6	BFHPC0.9	BFHPC1.2	BFHPC1.5	BFHPC1.8
Experimental	101.43	92.78	92.68	102.3	97.06	95.68
LR	98.1	97.1	96.6	96.1	95.6	95.1
SVR	96.7	94.9	95.4	96.3	96.8	96.2
PR	101	93	92.8	102	97	95.7

**Table 6:** The experimental and estimated flexural strength values (MPa).

Flexural Strength	HPC	BFHPC0.6	BFHPC0.9	BFHPC1.2	BFHPC1.5	BFHPC1.8
Experimental	14	15.6	17.4	18.9	18.1	18.3
LR	14.4	16.1	16.95	17.8	18.65	19.5
SVR	15.8	16.3	17.28	17.9	18.3	18
PR	13.9	15.5	17.5	18.75	18.2	18.25

**Table 7:** The experimental and estimated tensile strength values (MPa).

Tensile Strength	HPC	BFHPC0.6	BFHPC0.9	BFHPC1.2	BFHPC1.5	BFHPC1.8
Experimental	5.53	5.3	5.29	5.56	5.41	5.37
LR	5.45	5.43	5.42	5.51	5.4	5.39
SVR	5.425	5.368	5.41	5.46	5.51	5.47
PR	5.52	5.31	5.29	5.55	5.43	5.38

**Table 8:** Comparison of ME values (GPa).

ME	HPC	BFHPC0.6	BFHPC0.9	BFHPC1.2	BFHPC1.5	BFHPC1.8
Experiments	47.6	45.6	45.57	47.88	46.63	46.28
Prediction by LR	48.1	45.7	46.9	48.2	47	45.7
Prediction by SVR	46.6	46.2	46.3	46.5	46.6	45.5
Prediction by PR	47.6	45.7	45.6	47.7	46.7	46.4
ACI 318-08 [59]	48.02	45.8	46.23	47.88	47.43	45.8
Gardner and Lockman [60]	44.27	43.03	43.27	44.19	43.95	43.03
Eurocode [61]	47.11	45.11	45.49	46.99	46.6	45.11
CEB-FIP [62]	46.6	45.31	45.06	46.67	46.35	45.13

## Data Availability

Not applicable.
